# Enhancing Visible Light Photocatalytic Degradation of Bisphenol A Using BiOI/Bi_2_MoO_6_ Heterostructures

**DOI:** 10.3390/nano13091503

**Published:** 2023-04-28

**Authors:** Magaly Y. Nava Núñez, Moisés Ávila Rehlaender, Azael Martínez-de la Cruz, Arturo Susarrey-Arce, Francisco Mherande Cuevas-Muñiz, Margarita Sánchez-Domínguez, Tania E. Lara-Ceniceros, José Bonilla-Cruz, Alejandro Arizpe Zapata, Patricia Cerda Hurtado, Michael Pérez-Rodríguez, Aldo Ramírez Orozco, Lucy T. González, Francisco Enrique Longoria-Rodríguez

**Affiliations:** 1 Centro de Investigación en Materiales Avanzados SC, Subsede Monterrey, Alianza Norte 202, Apodaca 66628, NL, Mexico; magaly.nava@cimav.edu.mx (M.Y.N.N.); moises.avila@cimav.edu.mx (M.Á.R.); margarita.sanchez@cimav.edu.mx (M.S.-D.); tania.lara@cimav.edu.mx (T.E.L.-C.); jose.bonilla@cimav.edu.mx (J.B.-C.); alejandro.arizpe@cimav.edu.mx (A.A.Z.); patricia.cerda@cimav.edu.mx (P.C.H.); 2 CIIDIT, Facultad de Ingeniería Mecánica y Eléctrica, Universidad Autónoma de Nuevo León, Ciudad Universitaria, San Nicolás de los Garza 66451, NL, Mexico; azael70@yahoo.com.mx; 3 Mesoscale Chemical Systems, MESA+ Institute, University of Twente, Drienerlolaan 5, 7522 NB Enschede, The Netherlands; a.susarreyarce@utwente.nl; 4 Centro de Investigación y Desarrollo Tecnológico en Electroquímica, Parque Tecnológico Querétaro, s/n, Sanfandila, Pedro Escobedo 76703, QT, Mexico; fcuevas@cideteq.mx; 5 Tecnologico de Monterrey, Escuela de Ingeniería y Ciencias, Ave, Eugenio Garza Sada 2501 Sur, Monterrey 64890, NL, Mexicoaldo.ramirez@tec.mx (A.R.O.)

**Keywords:** BiOI, Bi_2_MoO_6_, BiOI/Bi_2_MoO_6_ heterostructures, photocatalysis, Bisphenol A

## Abstract

With the growing population, access to clean water is one of the 21st-century world’s challenges. For this reason, different strategies to reduce pollutants in water using renewable energy sources should be exploited. Photocatalysts with extended visible light harvesting are an interesting route to degrade harmful molecules utilized in plastics, as is the case of Bisphenol A (BPA). This work uses a microwave-assisted route for the synthesis of two photocatalysts (BiOI and Bi_2_MoO_6_). Then, BiOI/Bi_2_MoO_6_ heterostructures of varied ratios were produced using the same synthetic routes. The BiOI/Bi_2_MoO_6_ with a flower-like shape exhibited high photocatalytic activity for BPA degradation compared to the individual BiOI and Bi_2_MoO_6_. The high photocatalytic activity was attributed to the matching electronic band structures and the interfacial contact between BiOI and Bi_2_MoO_6_, which could enhance the separation of photo-generated charges. Electrochemical, optical, structural, and chemical characterization demonstrated that it forms a BiOI/Bi_2_MoO_6_ p-n heterojunction. The free radical scavenging studies showed that superoxide radicals (O_2_•^−^) and holes (h^+^) were the main reactive species, while hydroxyl radical (•OH) generation was negligible during the photocatalytic degradation of BPA. The results can potentiate the application of the microwave synthesis of photocatalytic materials.

## 1. Introduction

Water is a fundamental resource for socioeconomic development, food production, energy, and the survival of human beings [1]. Around the world, several lakes, rivers, canals, and other water bodies are heavily polluted by industrial and domestic discharges without further treatment, contributing to water pollution of aquifer ecosystems most prominently found in developing countries [2,3,4,5]. The wastewater can contain toxic inorganic pollutants, non-biodegradable dyes, heavy metals, pharmaceutics, and endocrine-disrupting chemicals (EDCs) [6]. Pesticides, herbicides, hormones and steroids, additives in personal care products, and plasticizers belong to the EDCs family [7]. Bisphenol A (BPA) is among the most common EDC in our daily life [8]. BPA is a monomer used in the polymer and polycarbonate industries. Everyday products with BPA include plastic containers and other plastic bottles [9]. Furthermore, reports have demonstrated that BPA has also been detected in the air and soil [10]. 

Reducing BPA is crucial because even at low concentrations, BPA can cause reproductive damage, such as female fertility problems, cancer, among other diseases [11,12]. The removal efficiency of BPA by conventional methods has been below 10% [7]. Traditional wastewater treatment methods such as adsorption, reverse osmosis, and biological processes are insufficient to eliminate BPA from wastewater systems [13]. A way forward is the use of oxidation technologies. Among the advanced oxidation process (AOP), heterogeneous photocatalysis is an emerging remediation technology leading to mineralizing most hazardous pollutants in water [14]. Key factors during the visible light activation of a photocatalyst are stability, recyclability, and efficiency for the degradation of organic compounds (e.g., BPA) until their complete mineralization (water and CO_2_) without forming secondary toxic substances [15,16].

Recently, bismuth-based compounds such as Bi_2_O_3_, BiVO_4_, Bi_2_WO_6_, Bi_2_MoO_6_, and BiOX (X = Cl, Br, I) have attracted attention due to their unique characteristics such as a layered architecture with strong visible-light absorption and high chemical stability [17]. Bismuth molybdate Bi_2_MoO_6_ has shown high performance for photocatalytic pollutant degradation in wastewater [18]. Nevertheless, applications of Bi_2_MoO_6_ in photocatalysis remain challenging due to the low active sites and relatively fast recombination rates of photo-generated electrons and holes that lead to reduced quantum yields [19,20]. From this perspective, Bi_2_MoO_6_, known for its *n*-type semiconductor properties, can be coupled to a *p*-semiconductor, forming an *n*-*p* heterostructure to extend the light absorption range and leverage the separation of photo-induced charges to participate in the reduction and oxidation reactions [21].

In this sense, *n-p* heterojunctions can be designed to improve the heterostructure efficiency. This is the case for heterostructures composed of Bi_2_MoO_6_ and *p*-type semiconductors, such as bismuth oxyiodide (BiOI), to enhance visible light absorption and the charge transfer process [22]. Among BiOX (X = I, Br, and C), BiOI has attracted quite some interest due to its narrower band gap and strong absorption of visible light irradiation [23]. Some authors reported a synergic effect between contacted BiOI/Bi_2_MoO_6_ heterostructures [24,25]. The proposed mechanism for this type of heterostructure is related to a built-in electronic field (BIEF) that can be created near the interfaces of *n*-*p* heterojunctions, which will be beneficial to promote charge carrier transfer, improving photocatalytic performance [26].

There are no reports addressing the synthesis of BiOI/Bi_2_MoO_6_ via microwave. The advantages of microwave synthesis are reaction time reduction, high reproducibility, enhancement in reaction yields, and the obtention of a more uniform product in dimensions and composition [27,28]. Yan et al. [24] reported for the first time the synthesis of BiOI/Bi_2_MoO_6_ using a two-step approach. In this work, Bi_2_MoO_6_ was synthesized via a solvothermal reaction, followed by heterostructure formation (i.e., BiOI/Bi_2_MoO_6_) by introducing BiOI. The results demonstrated that a BiOI/Bi_2_MoO_6_ heterostructure prepared with 25% BiOI content exhibited the highest photocatalytic activity for methylene blue and BPA degradation more than the individual BiOI and Bi_2_MoO_6_. Li et al. [29] prepared Bi_2_MoO_6_ by a solvothermal method, and in a second step, they incorporated the BiOI via coprecipitation synthesis. In this case, the BiOI/Bi_2_MoO_6_ heterostructure prepared with 30% BiOI presented the highest photocatalytic activity for rhodamine B degradation. The results reported that the enhanced photocatalytic activity of BiOI/Bi_2_MoO_6_ is mainly attributed to the formation of the *p-n* heterojunctions that can facilitate the separation and transfer of the photo-generated charge carriers. 

However, BiOI/Bi_2_MoO_6_ heterostructures with a lower proportion of the Bi_2_MoO_6_ have not been assessed yet. A lower amount of Bi_2_MoO_6_ can reduce the amount of Mo precursor, allowing the synthesis of a more sustainable material with lower Mo content. Motivated by this fact, in this work, we report an easy two-step microwave irradiation method to prepare BiOI/Bi_2_MoO_6_ heterostructures with low Bi_2_MoO_6_ content (5 and 10 wt%). The optical bandgap revealed that the BiOI/Bi_2_MoO_6_ heterostructures display a significant red-shift advancement compared with Bi_2_MoO_6_ due to the strong light-harvesting property of BiOI. Structural properties with XRD and TEM suggested the formation of a BiOI/Bi_2_MoO_6_ heterostructure. Furthermore, XPS and Raman confirmed the heterojunction between BiOI and Bi_2_MoO_6_. Electrochemical characterization using EIS demonstrates an improvement in the charge separation efficiency of the BiOI/Bi_2_MoO_6_ heterostructure with respect to individual BiOI. The functionality of the BiOI/Bi_2_MoO_6_ heterostructures was demonstrated during the photocatalytic degradation of BPA with degradation of ~90% under visible light irradiation.

## 2. Experimental Section 

### 2.1. Chemicals

The chemical reagents used in this study were bismuth nitrate pentahydrate (Bi(NO_3_)_3_·5H_2_O, ≥99%) and sodium molybdate (Na_2_MoO_4_·2H_2_O, 99.5%) from Sigma Aldrich (Merck KGaA, Darmstadt, Germany), potassium iodide (KI, 99.5%) and ethanol (99.6%) from DEQ (Garcia, NL, Mexico) and ethylene glycol (C_2_H_6_O_2_, 99.1%) from CTR (Monterrey, NL, Mexico). All reagents were used directly without any further purification.

### 2.2. Synthesis of Bi_2_MoO_6_

The Bi_2_MoO_6_ sample was prepared via microwave-assisted solvothermal synthesis. The procedure implied the preparation of two 0.1 M aqueous dissolutions of inorganic salts Bi(NO_3_)_3_·5H_2_O and Na_2_MoO_4_·2H_2_O in ethylene glycol. The molybdate solution was added by dropping it into the bismuth nitrate solution with a Bi/Mo molar ratio of 2:1. The solutions were mixed together with vigorous stirring at room temperature for 5 min to promote homogenization. Then, the resulting solution was transferred into a microwave glass vial. The microwave synthesis reaction was performed by increasing the temperature as fast as possible from 25 to 160 °C at a power of 800 W and the solution was held at this temperature for 1 h under continuous magnetic stirring at 800 rpm. Once the reaction time elapsed, the dispersion was cooled to 35 °C. After that step, the synthesized powders were separated from the ethylene glycol solution using centrifugation at a speed of 9000 rpm for 10 min. The product was washed three times with distilled water and two times with ethanol and dried in an electrical oven at 70 °C. Finally, the samples were calcinated in an electrical oven at 400 °C for 6 h.

### 2.3. Synthesis of BiOI and BiOI/Bi_2_MoO_6_

The BiOI pure sample was synthesized according to [29]. The method involved the preparation of two 0.1 M dissolutions of Bi(NO_3_)_3_·5H_2_O and KI in ethylene glycol. A stoichiometric amount of potassium iodide solution was added drop by drop into a nitrate solution to a complete volume of 20 mL. The resulting solution was collocated in a microwave reactor and maintained at 125 °C for 15 min.

BiOI/Bi_2_MoO_6_ heterostructures with a molar ratio of Bi_2_MoO_6_ (Mo/I, 5 and 10%) were prepared under the same above microwave-assisted solvothermal conditions for pure BiOI. However, in this case, firstly the dissolution of Bi(NO_3_)_3_·5H_2_O was added and subsequently, the as-prepared Bi_2_MoO_6_ powder and then KI solution was added. The resulting solution was collocated in a microwave reactor and maintained at 125 °C for 15 min. The final products were collected by centrifugation and washed three times with distilled water and two times with ethanol and dried in an electrical oven at 70 °C.

### 2.4. Characterization

The crystalline structure of the samples was analyzed via X-ray powder diffraction (XRD) using a Phillips X’Pert-Pro X-ray diffractometer with Cu Kα (λ = 1.5406 Å) radiation over a 2θ angle from 10 to 80° in steps of 0.033°/59.7 s. The obtained diffractograms were compared with those reported in the JCPDS Database. Raman spectroscopy analysis was carried out using a Raman spectrometer (Thermo Scientific DRX, Horiba Scientific™ Lab RamH Evolution Raman microscope, Darmstadt, Germany) with an excitation laser of 532 nm. The Raman spectra of the samples were acquired in the range of 100–1000 cm^−1^. The surface composition and elemental chemical states were investigated using X-ray photoelectron spectroscopy (XPS, Thermo Scientific, model Escalab 250Xi) with Al Kα X-rays (1486.68 eV). All the measurements were realized under an ultra-high vacuum (10^−10^ torr). The UV-vis reflection spectra were obtained using a UV–vis spectrophotometer (Agilent Technologies, model Cary 5000, Santa Clara, CA, USA) equipped with an integrating sphere assembly. The data were analyzed using the Kubelka–Munk function. The morphology, microstructure, and particle size of the samples were characterized using a scanning electron microscope (FEI Nova NanoSEM200, Hillsboro, OR, USA) and transmission electron microscope (TEM JEOL JEM 2200FS+CS, FS, USA). The Brunauer–Emmett–Teller (BET) specific surface area was obtained by measuring the N_2_ adsorption–desorption with an analyzed Bel-Japan Minisorp II after degassing the samples under vacuum at 100 °C for 24 h. The photoluminescence (PL) spectra were recollected at room temperature using a fluorescent spectrophotometer (Perkin Elmer LS55, Waltham, MA, USA). The emission spectra were acquired in the range of 400–600 nm by using an excitation wavelength of 400 nm.

### 2.5. Photocatalytic Activity

The photocatalytic activity of BiOI and BiOI/Bi_2_MoO_6_ heterostructures were examined for the degradation of BPA under visible light. The experimental test was conducted in a Batch photocatalytic reactor (250 mL capacity) equipped with a circulating water system. A total of 200 mg of the photocatalyst was dispersed in 200 mL of BPA solution (8 mg·L^−1^). Prior to illumination, the suspension was stirred for 60 min in the dark to reach the adsorption–desorption equilibrium. After this time, the sample was irradiated under visible illumination using an LED lamp (Street Light, 24 W) as a light source. At each time interval, 6 mL of solution was withdrawn and filtered by 0.22 μm PTFE filters. The concentration of BPA in the filtrate solution was monitored through the absorbance of its characteristic band at 276 nm using a UV-vis spectrophotometer (Agilent Technologies, Cary 5000 model). The BPA photodegradation of the samples was calculated using the following equation:

$$BPA\;degradation\;\left(\%\right)=\frac{C_{0}-C}{C_{0}}*100$$

where C_0_ represents the initial concentration and C is the final concentration of BPA after an irradiation time of 3 h. For trapping experiments, potassium iodide (KI, 99.5%) from DEQ, isopropanol (IPA, 99.5%), and p-benzoquinone (pBQ ≥ 98%) from Sigma Aldrich were used as radical scavengers to remove holes (h^+^), hydroxyl (•OH), and superoxide radicals (O_2_•^−^). The concentrations of scavenger KI, IPA, and pBQ in the solution were 0.4, 10, and 0.4 mM, respectively. The degraded percentage of BPA in the presence of each scavenger was estimated by analyzing the concentration via UV-vis spectrophotometers in the above-mentioned photocatalytic test.

## 3. Results

BiOI/Bi_2_MoO_6_ was synthesized using the microwave-synthesis route. The synergy between the BiOI/Bi_2_MoO_6_ heterostructure components was investigated structurally, chemically, and optically. The application of the BiOI/Bi_2_MoO_6_ heterostructure was assessed during the photocatalytic degradation of BPA and contrasted with BiOI and Bi_2_MoO_6_. A mechanism was proposed for BiOI/Bi_2_MoO_6_ degradation.

### 3.1. BiOI/Bi_2_MoO_6_ Heterostructure Synergy

#### 3.1.1. Structural Analysis of BiOI/Bi_2_MoO_6_

**Figure 1:**
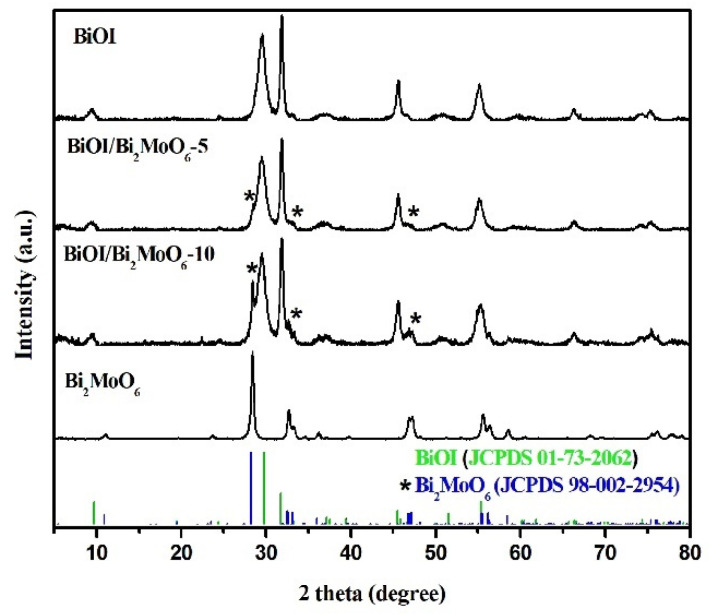
XRD patterns of Bi_2_MoO_6_, BiOI, and BiOI/Bi_2_MoO_6_ with different Bi_2_MoO_6_ concentrations. (* Reflections corresponding to the Bi_2_MoO_6_ phase. Blue lines: reflections corresponding to JCPDS standard No. 98-002-2954 corresponding to Bi_2_MoO_6_ and green lines: reflections corresponding to JCPDS standard No. 01-73-2062 corresponding to BiOI).

Figure 1 shows the DRX pattern of the samples Bi_2_MoO_6,_ BiOI, and BiOI/Bi_2_MoO_6_. The main diffraction lines of Bi_2_MoO_6_ were detected at 2θ = 10.8°, 28.5°, 32.4°, 33.0°, 36.0°, 46.7°, 47.0°, 55.4°, 56.1°, 58.4°, and 75.9°, which were indexed to the orthorhombic phase Bi_2_MoO_6_ according to the JCPDS card no. 98-002-2954. From the BiOI pattern, the diffraction peaks at 29.7°, 31.7°, 45.5°, 55.3°, 66.3°, 74.3°, and 75.4° in 2θ were assigned to the tetragonal phase of BiOI (JCPDS card no. 01-73-2062). No additional peaks were observed, confirming the purity of both photocatalysts. For the BiOI/Bi_2_MoO_6_-5 sample, no apparent diffraction lines of Bi_2_MoO_6_ were observed, possibly due to the low amount of Bi_2_MoO_6_ in the heterostructure; however, when the molar ratio Mo/I increased from 5 to 10%, an important peak was observed at around 28° in 2θ. This diffraction peak corresponds to the (131) plane of Bi_2_MoO_6_, according to [30]. Therefore, these results suggest the successful formation of the BiOI/Bi_2_MoO_6_ heterostructures. 

**Figure 2:**
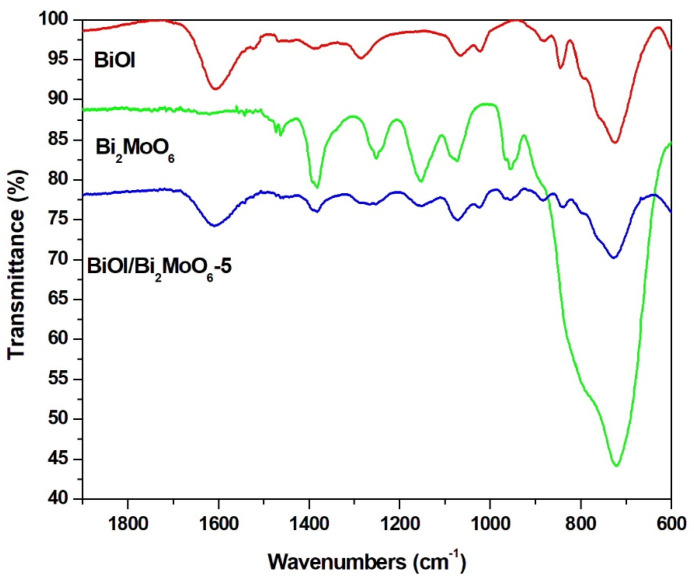
FT−IR spectra of BiOI, Bi_2_MoO_6_, and BiOI/Bi_2_MoO_6_-5 heterostructure.

The FT-IR analysis of BiOI, Bi_2_MoO_6_, and BiOI/Bi_2_MoO_6_ samples is shown in Figure 2. In all the samples, the presence of one characteristic band was detected at about 1460 cm^−1^ corresponding to the O–H moiety emanating from water and ethylene glycol. For BiOI pure, the peak at a low frequency of about 500 cm^−1^ is attributed to the vibration of Bi–O chemical bonds in BiOI, which also can be found in the BiOI/Bi_2_MoO_6_ heterostructure. On the other hand, absorption peaks at 728 and 798 cm^−1^ corresponded to the stretching vibration Mo–O bond peak in Bi_2_MoO_6_. Meanwhile, the FT-IR result of the BiOI/Bi_2_MoO_6_ sample indicates that the heterostructure contains two fundamental components, BiOI and Bi_2_MoO_6_.

**Figure 3:**
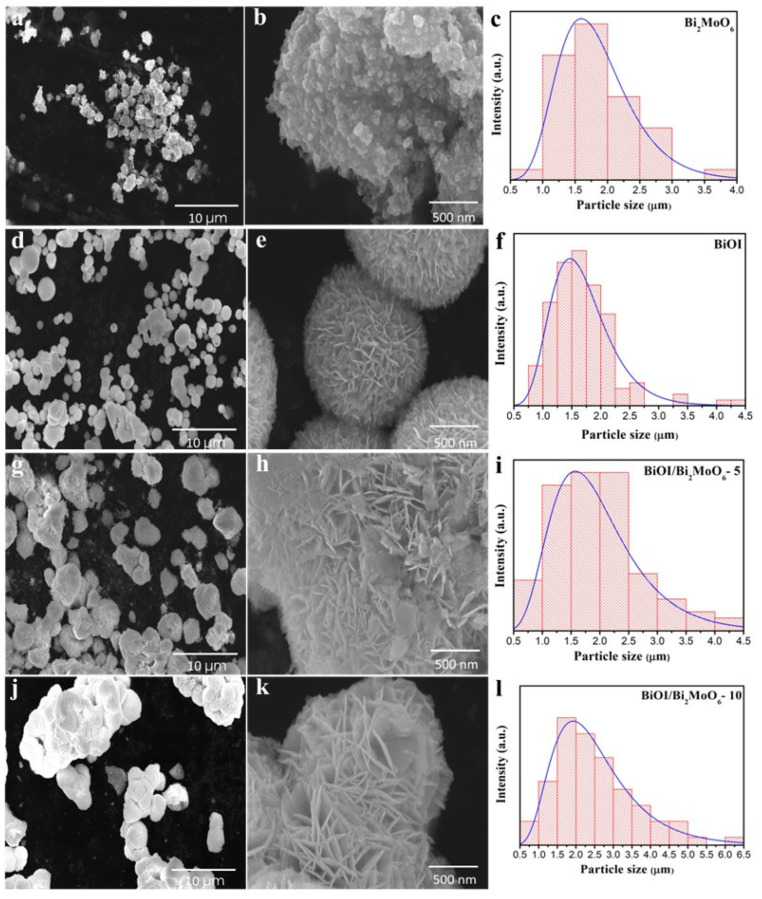
SEM images and distribution particle size of Bi_2_MoO_6_ (**a**–**c**), BiOI (**d**–**f**), BiOI/Bi_2_MoO_6_-5 (**g**–**i**), and BiOI/Bi_2_MoO_6_-10 (**j**–**l**).

Figure 3 shows the scanning electron microscopy (SEM) images of the pure Bi_2_MoO_6_, BiOI, and heterostructure BiOI/Bi_2_MoO_6_ synthesized by microwave-assisted synthesis. In the low magnification SEM image presented in Figure 3a, it can be seen that the pure Bi_2_MoO_6_ sample is composed of a non-uniform morphology with shape and variable particle size. From the high magnification SEM image, it can be observed that this sample was comprised of the attachment of many irregular nanoparticles with a particle size of ~30–40 nm, giving them a scaly appearance (Figure 3b). The histogram presented in Figure 3c shows that the average particle size of pure Bi_2_MoO_6_ was 1.84 μm. On the other hand, as is shown in Figure 3d, distinctive differences between the morphology and particle size of Bi_2_MoO_6_ and BiOI were observed. Pure BiOI presented a flower-like microsphere morphology with a diameter size of 1 to 2.5 μm (Figure 3d). The high-magnification SEM image illustrates that the microspheres were constructed by the self-assembly of plentiful smooth and ultrathin nanosheets with a thickness of ~5 nm. The nanosheets seem highly organized and assembled from the center to the surface of the microspheres. No insolated nanosheets were observed (Figure 3e). The average particle size distribution for BiOI was 1.68 μm, as can be seen in Figure 3f. After the heterojunction between the Bi_2_MoO_6_ and BiOI photocatalysts, the morphologies of the BiOI/Bi_2_MoO_6_ heterostructures significantly changed. With increases of 5 and 10% of Bi_2_MoO_6,_ the flowering structure of the BiOI sample gradually disappears because the Bi_2_MoO_6_ serves as a supporting platform for the growth of BiOI. In some specific zones, the BiOI gradually grows on the surface of the Bi_2_MoO_6_ particles and becomes less compact in microspheres. In other zones, the nanosheets of BiOI covered the surface of Bi_2_MoO_6_ completely. The high-magnification SEM image showed an intimate interface contact between Bi_2_MoO_6_ and BiOI particles, which is ideal for effective charge transfer between both photocatalysts and could contribute to enhancements in the photocatalytic activity. As can be seen in the histograms presented in Figure 3i,j, the BiOI/Bi_2_MoO_6_-5 sample displayed a smaller particle size (1.97 μm) among the heterostructures than the BiOI/Bi_2_MoO_6_-10 sample (2.54 μm). On the other hand, in the EDS spectrum of BiOI/Bi_2_MoO_6_-5 (see Figure S1), the corresponding lines of Bi, I, Mo, and O can be observed, suggesting the possible formation of the heterojunction of BiOI and Bi_2_MoO_6_.

**Figure 4:**
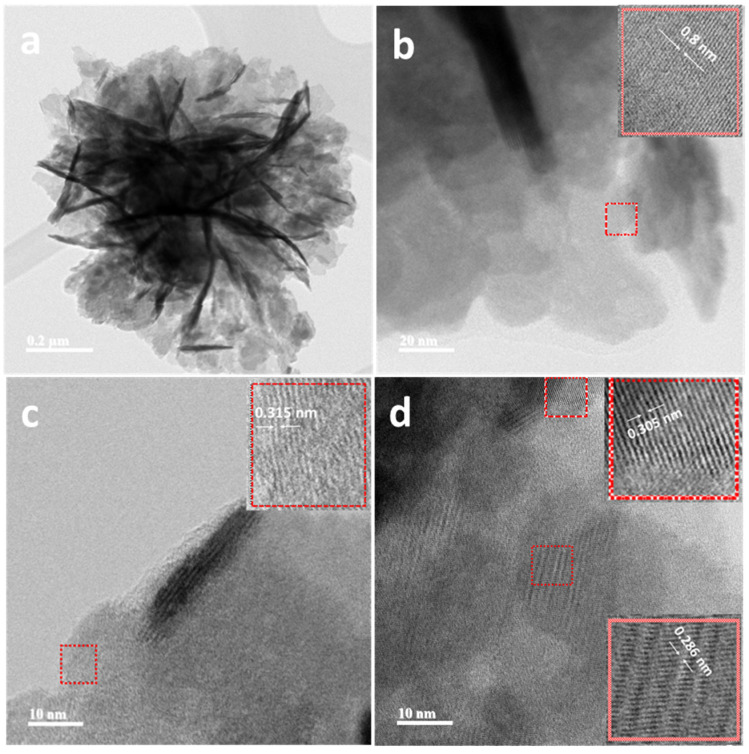
TEM and HRTEM images of the BiOI/Bi_2_MoO_6_-5 sample. BiOI/Bi_2_MoO_6_-5 heterostructure microsphere (**a**); d-spacing of 0.8 and 0.315 nm corresponding to the (020) and (131) planes of Bi_2_MoO_6_ d spacing of (**b**,**c**); 0.286 and 0.305 corresponding to the (110) and (012) planes of BiOI (**d**).

TEM and HRTEM further characterized the BiOI/Bi_2_MoO_6_-_5_ heterostructure. The distinct lattice fringes observed in Figure 4 with an interval of 0.8 and 0.315 nm correspond to the (020) and (131) planes of Bi_2_MoO_6_. Furthermore, the d spacing of 0.286 and 0.305 agreed well with the (110) and (012) planes of BiOI. This fact also suggests that Bi_2_MoO_6_ was successfully combined with BiOI. The heterojunction interface between both photocatalysts could accelerate the separation of photo-generated charges.

#### 3.1.2. Chemical Species in BiOI/Bi_2_MoO_6_

**Figure 5:**
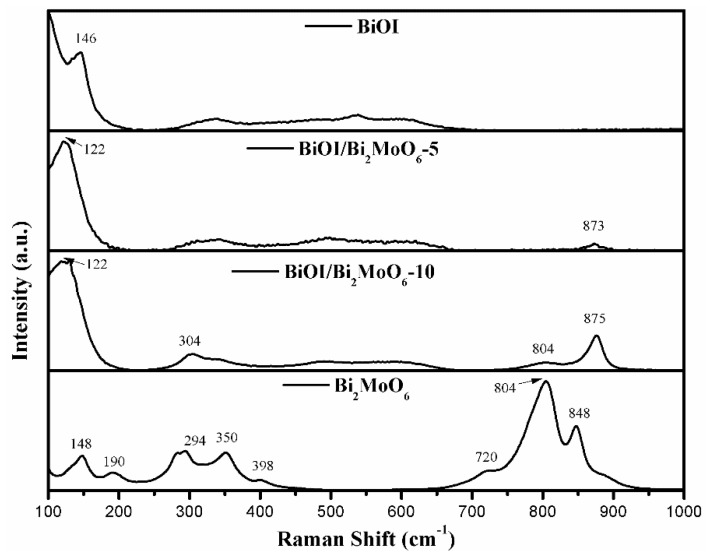
Raman spectra of Bi_2_MoO_6,_ BiOI, BiOI/Bi_2_MoO_6_-5, and BiOI/Bi_2_MoO_6_-10.

The room-temperature Raman spectrum of BiOI, BiOI/Bi_2_MoO_6_-5, BiOI/Bi_2_MoO_6_-10, and Bi_2_MoO_6_ are presented in Figure 5. In the BiOI spectrum, the main peak at 146 cm^−1^ was attributed to the stretching mode of Bi-I [31,32]. In the Bi_2_MoO_6_ spectrum, the peaks from 848–720 cm^−1^ and 398–294 cm^−1^ were attributed to stretching and bending modes of the MoO_6_ octahedron, respectively, while the 190 and 148 cm^−1^ peaks correspond to vibration modes of the [Bi_2_O_2_]^2+^ framework [26]. In the case of BiOI/Bi_2_MoO_6_-5, it is possible to observe the appearance of a weak peak at 873 cm^−1^, which can be assigned to the Mo-O stretching of MoO_6_. The fact that the peak was significantly displaced towards a higher wavenumber value indicates a strong interface contact between Bi_2_MoO_6_ and BiOI, confirming the formation of the heterostructure. The same case occurs for BiOI/Bi_2_MoO_6_-10, where the peak appears to shift to 875 cm^−1^ with a stronger intensity than BiOI/Bi_2_MoO_6_-5, indicating a higher content of the Bi_2_MoO_6_ phase, and additionally, signals typical of the Bi_2_MoO_6_ phase appeared at 804 cm^−1^ and 304 cm^−1^. In previous reports, a peak shift of a vibrational mode in a Raman spectrum has been observed due to the interfacial interaction between two phases of a heterostructure [33].

### 3.2. X-ray Photoelectron Spectroscopy

**Figure 6:**
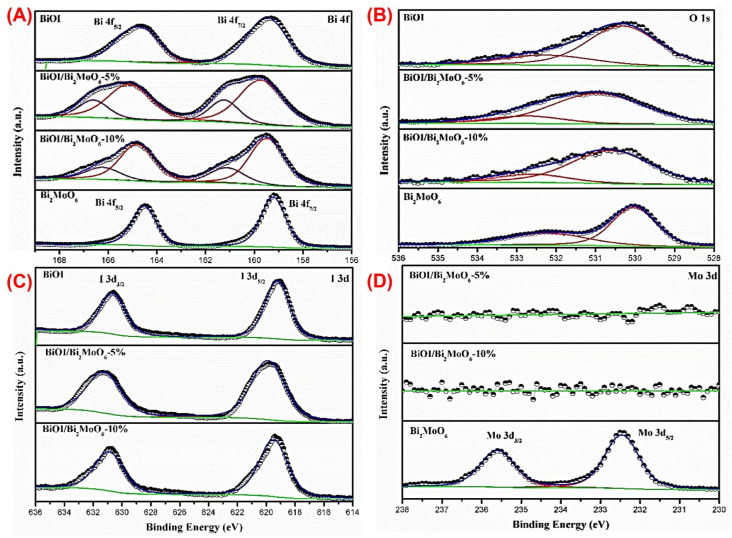
XPS spectra of BiOI, Bi_2_MoO_6_, BiOI/Bi_2_MoO_6_-5 and BiOI/Bi_2_MoO_6_-10, (**A**) Bi 4f, (**B**) O 1s, (**C**) I 3d, and (**D**) Mo 3d. Spectral signal of Bi, O, I and Mo (blue line); contributors to the spectral signal of each element (red lines); background (green line).

The elemental composition and surface chemical states of BiOI, BiOI/Bi_2_MoO_6_-5, BiOI/Bi_2_MoO_6_-10, and Bi_2_MoO_6_ were obtained by XPS. Figure 6 shows the full scan of XPS survey spectra. In BiOI the presence of Bi, O, and I was detected, as well as in the BiOI/Bi_2_MoO_6_-5 and BiOI/Bi_2_MoO_6_-10 heterostructures, although, for these samples, the presence of Mo was not detected. In Bi_2_MoO_6_ also, all chemical elements were detected. Figure 6 shows the high-resolution XPS spectra of Bi 4f, O 1s, I 3d, and Mo 3d. The C 1s peak (284.8 eV) was used to correct the binding energy values of all elements. A summary of XPS results for BiOI, BiOI/Bi_2_MoO_6_-5, BiOI/Bi_2_MoO_6_-10, and Bi_2_MoO_6_ is shown in Table 1. 

For BiOI, the binding energies around 159.3 and 164.7 eV correspond to the signals from the doublets of Bi 4f_7/2_ and Bi 4f_5/2_ which suggests a trivalent oxidation state for Bi. Meanwhile, for BiOI/Bi_2_MoO_6_-5, a significant broadening of the Bi 4f signals was resolved in a deconvulsion; the first doublet was obtained at 159.8 and 165.2 eV corresponding to the Bi 4f_7/2_ and Bi 4f_5/2_ orbitals. In comparison, the second doublet was displaced to higher BE values with 161.3 and 166.6 eV corresponding to the Bi 4f_7/2_ and Bi 4f_5/2_ orbitals with a different chemical environment than the first ones. The same behavior was observed for BiOI/Bi_2_MoO_6_-10, where the binding energy of Bi 4f shifted to higher values compared with that of Bi 4f of pure BiOI at 159.5 and 161.2 (Bi 4f_5/2_) and 164.8 and 166.3 eV (Bi 4f_7/2_). In the case of heterostructures, there is the possibility that Mo 3d has not been detected because it is totally masked by the growth of BiOI over the entire surface of Bi_2_MoO_6_, such as was observed in SEM images.

#### Optical Properties of the BiOI/Bi_2_MoO_6_ Heterostructure Components

**Figure 7:**
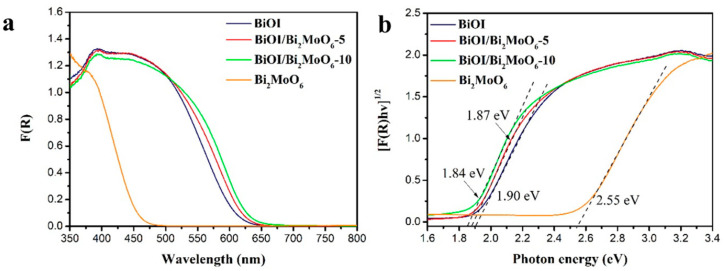
(**a**) Kubelka–Munk absorption curves and (**b**) plots of (FRhv)^1/2^ vs. photon energy (hv) for Bi_2_MoO_6,_ BiOI, BiOI/Bi_2_MoO_6_-5, and BiOI/Bi_2_MoO_6_-10.

The optical properties of the samples were investigated using UV-visible diffuse reflectance. The absorption spectra of Bi_2_MoO_6_, BiOI, and BiOI/Bi_2_MoO_6_ were determined from reflectance data using the Kubelka–Munk equation. From Figure 7a it can be observed that the BiOI exhibited a strong light absorption in the visible range at an absorption edge of about 600 nm. The absorption edge for the Bi_2_MoO_6_ sample was about 450 nm, indicating less response to visible light. On the other hand, the absorption threshold of the BiOI/Bi_2_MoO_6_ heterojunctions was significantly red-shifted compared to Bi_2_MoO_6_. BiOI/Bi_2_MoO_6_-10 displayed higher absorption in the visible light region, indicating decreased energy band gap. This behavior can be due to heterojunction formation in the interface of Bi_2_MoO_6_ and BiOI, which improves the efficiency of the photo-excited electrons.

The values of energy bandgap (E_g_) were calculated according to the Tauc plot, extrapolating the linear region of [FR(hv)]^1/2^ on the *y*-axis versus photon energy (hν) on the *x*-axis (see, Figure 7b). The energy bandgaps (E_g_) calculated for Bi_2_MoO_6_, BiOI, BiOI/Bi_2_MoO_6_-5, and BiOI/Bi_2_MoO_6_-10 were 2.54, 1.90, 1.87, and 1.84 eV, respectively. It can be seen that the optical band gap of the heterostructures is found in values closer to BiOI because they are in greater proportion than Bi_2_MoO_6_. The BiOI/Bi_2_MoO_6_-10 sample presented the narrowest energy band gap among the samples, which could be beneficial in improving the photocatalytic activity because more photo-generated charges could participate in the photocatalytic process. Thus, these BiOI/Bi_2_MoO_6_ heterostructures may be ideal visible-light-driven photocatalysts to expand the optical response to the visible light region compared with TiO_2_ and ZnO.

### 3.3. Heterostructure Synergy to Promote Photocatalytic Degradation

**Figure 8:**
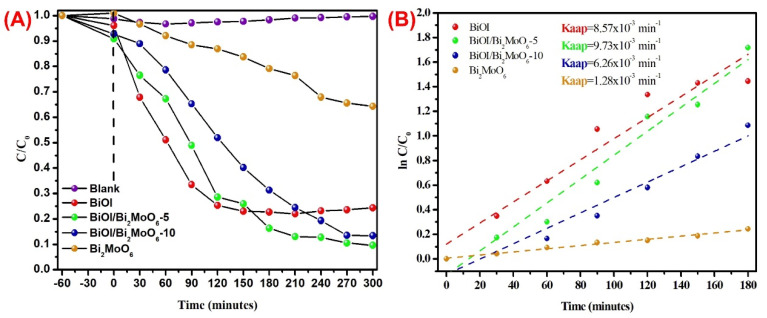
(**A**) Plot of C/C_0_ versus time and (**B**) ln (C/C_0_) versus time for the degradation of BPA in the presence of Bi_2_MoO_6_, BiOI, BiOI/Bi_2_MoO_6_-5, and BiOI/Bi_2_MoO_6_-10 under visible light irradiation.

The degradation capabilities of as-prepared samples were evaluated under visible light illumination, considering the BPA as the target contaminant. Before irradiation, the photocatalytic system was stirred in the dark for 60 min to achieve the adsorption–desorption equilibriums between each photocatalyst and BPA solution. Likewise, direct BPA photolysis under visible light was performed. As depicted in Figure 8, individual BiOI and BiOI/Bi_2_MoO_6_ heterostructures showed a slightly higher adsorption capability for BPA than pure Bi_2_MoO_6_. However, the BPA absorbed on the surface of all as-synthesized samples was less than 10% under visible-light irradiation. The adsorption of each photocatalyst plays a certain role in the photocatalytic process and is favorable for degradation reactions. on the other hand, the blank experiment reveals that the self-photodegradation of BPA after 5 h was negligible, indicating that visible light irradiation possessed no photocatalytic effect on BPA pollutants, as is shown in Figure 8A. Among the samples, pure Bi_2_MoO_6_ showed poor photocatalytic activity, which leads to a degradation degree of 36%. Moreover, BiOI displayed relatively acceptable photocatalytic performance, which was 76% after 300 min. When heterostructured BiOI/Bi_2_MoO_6_ samples were tested, the photocatalytic behavior was greatly enhanced compared with individual Bi_2_MoO_6_ and BiOI. The degradation degrees of BPA when BiOI/Bi_2_MoO_6-_5 and BiOI/Bi_2_MoO_6-_10 were used as photocatalysts were 90 and 87%, respectively. 

**Figure 9:**
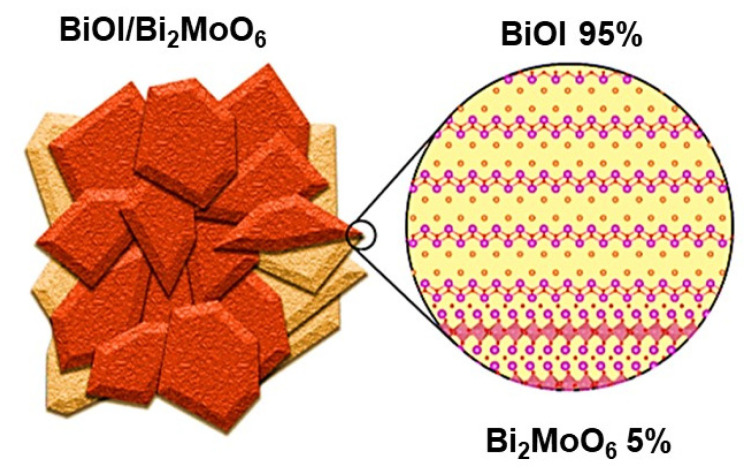
Scheme of the possible interface between the crystalline structures of BiOI and Bi_2_MoO_6_.

The corresponding photodegradation kinetics plot of BPA in the presence of the as-prepared samples is displayed in Figure 8B. As can be seen, the kinetics were adjusted up to 180 min since, after that time, the BiOI lost the behavior of pseudo-order one. Table 2 summarizes the rate constants obtained in the photocatalytic degradation of BPA. The results demonstrate that among all the samples, the BiOI/Bi_2_MoO_6_-5 heterostructure obtained the highest apparent constant *K*_app_ = 9.73 × 10^−3^ min^−1^, greatly higher than individual Bi_2_MoO_6_ and BiOI. The above results indicate that BiOI/Bi_2_MoO_6_-5 is more effective for BPA photodegradation. Therefore, the optimal percentage of Bi_2_MoO_6_ in the composite was 5 wt%. The high degradation of BPA by the BiOI/Bi_2_MoO_6_-5 sample could be attributed to the crystalline structure of both phases and the formation of a p-n-type heterojunction, which contributes to the stronger visible light absorption ability. Likewise, the BiOI/Bi_2_MoO_6_ heterojunction possesses favorable intimate contact that favors interfacial contact between both photocatalysts. The crystal structure of Bi_2_MoO_6_ and BiOI phases are Aurivillius type, described as a combination of a [Bi_2_O_2_]^2+^ layered sandwich between two MoO_4_^2−^ for Bi_2_MoO_6_ and a bilayer I^-^ ions in the case of BiOI. In the literature, it has been reported that the internal electrostatic fields between the positive layers of [Bi_2_O_2_]^2+^ and the anionic layers of Bi_2_MoO_6_ and BiOI can induce the effective separation of photo-generated charges and also form a narrower energy band gap. A representation of the BiOI/Bi_2_MoO_6_ heterostructure and the possible interface between both crystalline structures is presented in Figure 9.

**Figure 10:**
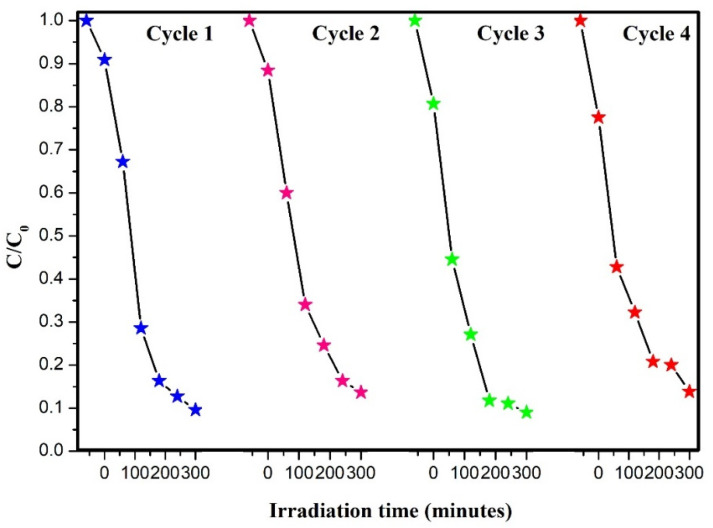
Cycling degradation experiment using the BiOI/Bi_2_MoO_6_-5 sample. Blue stars (cycle 1); magenta (cycle 2); green (cycle 3) and red (cycle 4).

The stability of the photocatalysts is an important factor that should be considered in the photocatalytic processes. To evaluate the stability of the photocatalyst, repeated photocatalytic tests were performed. For carrying out this experiment, the BiOI/Bi_2_MoO_6_-5 sample was selected. The sample was tested under the same photocatalytic conditions mentioned above. However, after each photocatalytic test, the photocatalyst was recovered by filtration and was sometimes washed with deionized water. The results presented in Figure 10 reveal that the photodegradation of BPA only slightly decreased after four successive cycles, demonstrating that the BiOI/Bi_2_MoO_6_-5 sample presented good long-term stability for BPA degradation. The stability of the solid after three degradation cycles was evaluated using FTIR and XRD techniques. As can be seen in Figure S2a, the bands of the IR spectra obtained do not present significant differences, likewise, no differences are observed in the reflections of the diffractograms of the heterostructure before and after photodegradation (see Figure S2b), due to which it can be assumed that the BiOI/Bi_2_MoO_6_-5 heterojunction is stable after several cycles of the degradation process. 

### 3.4. Mechanistic Insights of the BiOI/Bi_2_MoO_6_ Heterostructure

**Figure 11:**
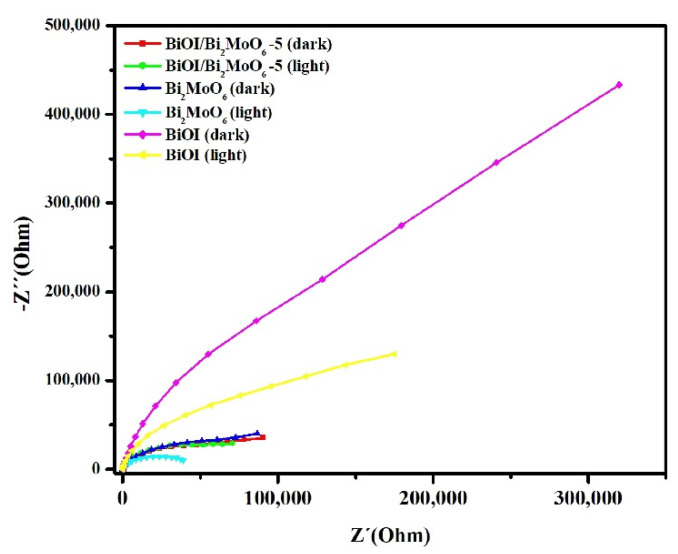
EIS Nyquist plot of BiOI, Bi_2_MoO_6,_ and BiOI/Bi_2_MoO_6_-5.

Electrochemical impedance spectroscopy (EIS) measurements were performed to investigate the effect of Bi_2_MoO_6_ on the photoelectric properties of BiOI/Bi_2_MoO_6_ heterostructures. Figure 11 displays the EIS Nyquist plots of pure BiOI, Bi_2_MoO_6_, and BiOI/Bi_2_MoO_6_-5. Under simulated solar irradiation, the BiOI/Bi_2_MoO_6_-5 sample displayed a smaller arc radius than pure BiOI, indicating the photo-generated charges’ high separation and transfer efficiency. Although Bi_2_MoO_6_ displayed a much-depressed arc radius compared to other samples, which could imply less interfacial resistance for charge transfer, other properties, such as specific surface area (SSA), may have significantly influenced the photocatalytic activity. In this case, the large specific surface area of BiOI (57 m^2^ g^−1^) compared with Bi_2_MoO_6_ (13 m^2^ g^−1^) could provide more active sites in the heterostructures, which greatly favored the photocatalytic activity of the BiOI and BiOI/Bi_2_MoO_6_ samples.

**Figure 12:**
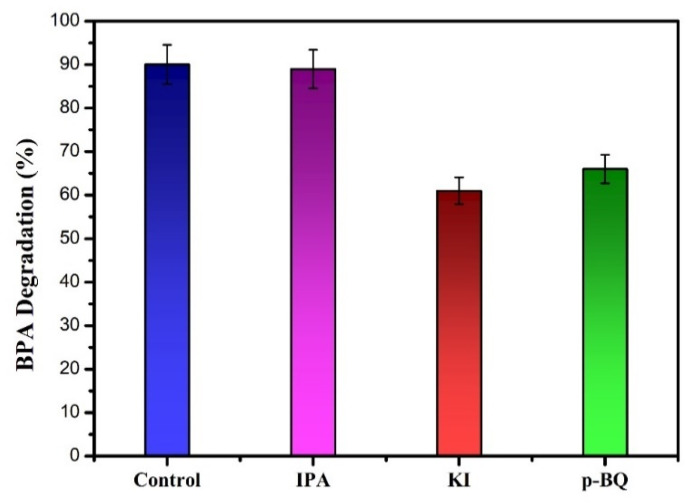
Trapping experiments using the BiOI/Bi_2_MoO_6_-5 sample.

Some active species, such as holes (h^+^), hydroxide (•OH), and superoxide radicals (O_2_•^−^) can be generated during the photocatalytic process under visible or UV light irradiation. To investigate the effect of the active species in the photocatalytic process for BPA degradation, a series of quencher substances were introduced to the photocatalytic system. For this purpose, isopropyl alcohol (IPA), potassium iodide (KI), and p-benzoquinone (BQ) were used for the respective hydroxide radical, hole, and superoxide radical trappings [34]. Figure 12 shows that BPA degradation under visible light irradiation was not affected by the addition of IPA, suggesting that hydroxyl radical (•OH) was a non-active species in the photocatalytic reaction. However, after the addition of p-BQ and KI, the photocatalytic BPA degradation was significantly reduced to 69 and 61%. These results suggest that superoxide radicals (O_2_•^−^) and holes (h^+^) are the main active species contributing to the photocatalytic process for BPA degradation using the BiOI/Bi_2_MoO_6_-5 sample.

In order to understand the photo-induced charge transfer and separation process in detail, the potentials of the conduction band (CB) and valence band (VB) edges BiOI and Bi_2_MoO_6_ were theoretically predicted by the following equations:

(1) $$E_{CB}=X-E_{C}-0.5E_{g}$$

(2) $$E_{VB}=E_{CB}+E_{g}$$

where $E_{CB}$ and $E_{VB}$ are the CB and VB edge potentials, X is the electronegativity of the semiconductor, $E_{C}$ is the energy of free electrons on the hydrogen scale (about 4.5 eV), and $E_{g}$ is the band gap energy of the semiconductor obtained by DRS measurements. The potential data of BiOI and Bi_2_MoO_6_ is listed in Table 2. The calculated results indicate that the band potentials of BiOI and Bi_2_MoO_6_ do not take on a staggered band alignment due to the energies of the photo-generated carriers being approximately equal. However, some reports indicate that the VB edge of BiOI could rise to a higher potential edge (−0.56 eV) under visible light [35,36]. When the BiOI/Bi_2_MoO_6_ heterostructure is exposed to visible light irradiation, the valence band electron of BiOI and Bi_2_MoO_6_ can be excited to the conduction band. The electrons in the uppermost valence band of *p*-type BiOI could jump into the conduction band and then transfer to the conduction band of *n*-type Bi_2_MoO_6_. Conversely, the holes in the valence band would flow in the opposite direction under the influence of the internal electrostatic field (from Bi_2_MoO_6_ to BiOI). The electrons can react with O_2_ adsorbed to produce O_2_•^−^. Additionally, although the •OH radicals could not be directly generated from BiOI since the standard redox potential is more negative than +2.72 eV (•OH/H_2_O) [36], it is known that it could not only form photo-generated holes but also photo-generated electrons. As a result, the recombination of electrons and holes is reduced, which has been confirmed by the above EIS analysis, where it was observed that the arc radius of the BiOI/Bi_2_MoO_6_-5 heterostructure was significantly smaller than individual BiOI.

## 4. Conclusions

In the present study, pure BiOI, Bi_2_MoO_6_, and BiOI/Bi_2_MoO_6_ samples were successfully prepared by microwave-assisted synthesis and characterized by several techniques. SEM images exhibited that the BiOI/Bi_2_MoO_6_ heterostructure was obtained with the growth of BiOI over the surface of Bi_2_MoO_6_. DRX, TEM, and XPS analysis revealed that an intimate interface between BiOI and Bi_2_MoO_6_ formed in the BiOI/Bi_2_MoO_6_ heterostructure. Compared with pure Bi_2_MoO_6_ and BiOI, the BiOI/Bi_2_MoO_6_ samples demonstrated superior photocatalytic activity for BPA degradation under visible light irradiation. Photocatalytic experiments indicated that BiOI/Bi_2_MoO_6_-5 exhibited the highest photocatalytic activity among all samples (~90%). The enhanced photocatalytic performance of BiOI/Bi_2_MoO_6_ heterostructures could be attributed to the *n-p* heterojunction formed between BiOI and Bi_2_MoO_6_, which not only expanded the range of the absorption spectrum to visible light but also improved the separation of photogenerated charges. Quencher experiments indicated that the holes and superoxide radicals were the predominant reactive species for the photocatalytic removal of BPA. Finally, the good stability demonstrated by the BiOI/Bi_2_MoO_6_-5 sample after repeated cycles suggests that this heterostructure may be proposed as a potential photocatalyst for environmental remediation.

## Figures and Tables

**Table 1:** Binding energies for the high-resolution XPS spectra of BiOI, BiOI/Bi_2_MoO_6_-5, BiOI/Bi_2_MoO_6_-10, and Bi_2_MoO_6_-5 samples.

Sample		Binding Energy (eV)						
	Relation Bi:I	Bi 4f_7/2_	Bi 4f_5/2_	I 3d_5/2_	I 3d_3/2_	Mo 3d_5/2_	Mo 3d_3/2_	O 1s
BiOI	0.89	159.3	164.7	619.2	630.7	-	-	530.3
BiOI/Bi_2_MoO_6_-5	0.87	159.8 161.3	165.2 166.6	619.8	631.3	-	-	531.3
BiOI/Bi_2_MoO_6_-10	0.84	159.5 161.2	164.8 166.3	619.5	630.9	-	-	530.7
Bi_2_MoO_6_	-	159.2	164.5	-	-	232.5	235.6	530.3

**Table 2:** Calculated E_CB_ and E_VB_ of BiOI and Bi_2_MoO_6_ photocatalysts.

Samples	*X* (eV)	Eg (eV)	*E*_CB_ (eV)	*E*_VB_ (eV)
BiOI	5.99	1.9	0.54	2.44
Bi_2_MoO_6_-5	5.5	2.55	−0.275	2.275

## Data Availability

Not applicable.

## Supplementary Materials

The following supporting information can be downloaded at: https://www.mdpi.com/article/10.3390/nano13091503/s1, Figure S1: (a) SEM micrograph and (b) EDS spectrum of BiOI/Bi_2_MoO_6_-5; Figure S2: (a) IR spectra and (b) diffractograms obtained from the BiOI/Bi_2_MoO_6_-5 heterostructure before and after various degradation processes.
