# Supplementary material for: Enhancing Visible Light Photocatalytic Degradation of Bisphenol A Using BiOI/Bi2MoO6 Heterostructures
Source: Nanomaterials (Basel). 2023 Apr 28;13(9):1503. doi: 10.3390/nano13091503 (PMC10179956; doi:10.3390/nano13091503)
Supplement: Supplementary file 1 [file nanomaterials-13-01503-s001.zip › nanomaterials-2332392-supplementary.pdf]

Supporting Information

For

Enhancing Visible Light Photocatalytic Degradation of Bisphenol A Using BiOI/Bi_2MoO_6 Heterostructures

Magaly Y. Nava Núñez [1], Moisés Ávila Rehlaender [1], Azael Martínez-de la Cruz [2], Arturo Susarrey-Arce [3], Francisco Mherande Cuevas-Muñiz [4], Margarita Sánchez-Domínguez [1], Tania E. Lara-Ceniceros [1], José Bonilla-Cruz [1], Alejandro Arizpe Zapata [1], Patricia Cerda Hurtado [1], Michael Pérez-Rodríguez [5], Aldo Ramírez Orozco [5], Lucy T. González [5,*] and Francisco Enrique Longoria-Rodríguez [1,*]

[1] Centro de Investigación en Materiales Avanzados SC, Unidad Monterrey, Alianza Norte 202,66628, Apodaca, NL, Mexico; magaly.nava@cimav.edu.mx (M.Y.N.N.); moises.avila@cimav.edu.mx (M.Á.R.); margarita.sanchez@cimav.edu.mx (M.S.D.); tania.lara@cimav.edu.mx (T.E.L.C.); jose.bonilla@cimav.edu.mx (J.B.C.), alejandro.arizpe@cimav.edu.mx (A.A.Z.); patricia.cerda@cimav.edu.mx (P.C.H.)

[2] CIIDIT, Facultad de Ingeniería Mecánica y Eléctrica, Universidad Autónoma de Nuevo León, Ciudad Universitaria, San Nicolás de los Garza 66451, Mexico; azael70@yahoo.com.mx

[3] Mesoscale Chemical Systems, MESA+ Institute, University of Twente, Drienerlolaan 5, 7522 NB, Enschede, The Netherlands; a.susarreyarce@utwente.nl

[4] Centro de Investigación y Desarrollo Tecnológico en Electroquímica, Parque Tecnológico Querétaro, s/n, Sanfandila, Pedro Escobedo, 76703, Qro., Mexico; fcuevas@cideteq.mx

[5] Tecnologico de Monterrey, Escuela de Ingeniería y Ciencias, Ave, Eugenio Garza Sada 2501 Sur, Monterrey 64890, NL, Mexico; aldo.ramirez@tec.mx (A.R.O.)

* Correspondence: lucy.gonzalez@tec.mx (L.T.G.); francisco.longoria@cimav.edu.mx (F.E.L.R.)

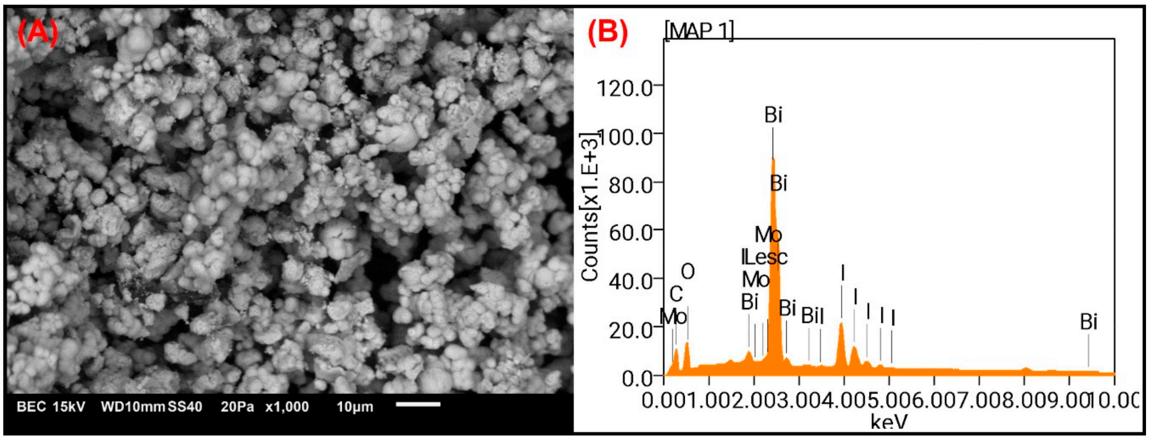

Figure S1. a) SEM micrograph and b) EDS spectrum of $BiOI/Bi_2MoO_6$-5

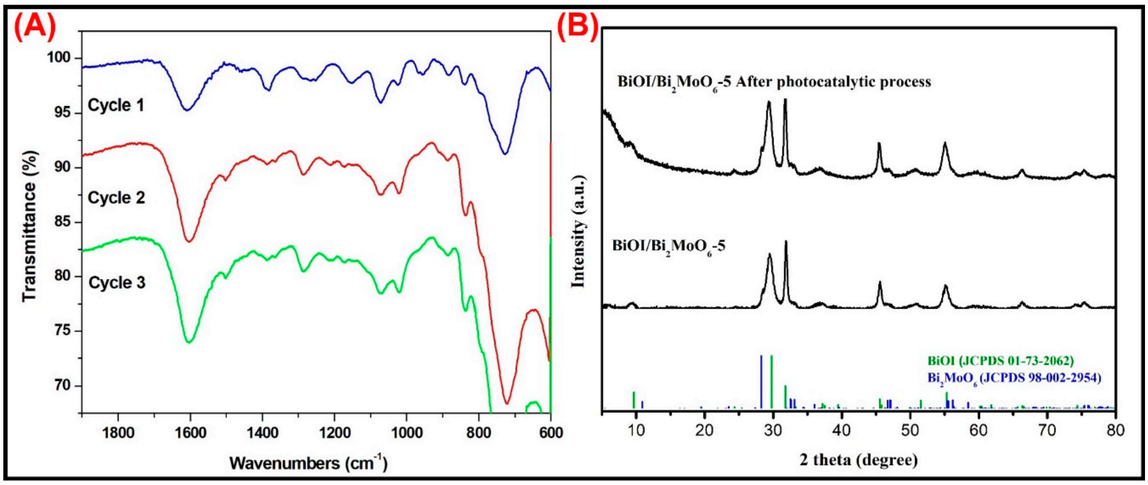

Figure S2. a) IR spectra and b) diffractograms obtained from the $BiOI/Bi_2MoO_6$-5 heterostructure before and after various degradation processes.
